# The structure of the Shiga toxin 2a A‐subunit dictates the interactions of the toxin with blood components

**DOI:** 10.1111/cmi.13000

**Published:** 2019-01-18

**Authors:** Maurizio Brigotti, Dorothea Orth‐Höller, Domenica Carnicelli, Elisa Porcellini, Elisabetta Galassi, Pier Luigi Tazzari, Francesca Ricci, Francesco Manoli, Ilse Manet, Heribert Talasz, Herbert H. Lindner, Cornelia Speth, Thomas Erbeznik, Stefan Fuchs, Wilfried Posch, Sneha Chatterjee, Reinhard Würzner

**Affiliations:** ^1^ Dipartimento di Medicina Specialistica, Diagnostica e Sperimentale, Sede di Patologia Generale Università di Bologna Bologna Italy; ^2^ Division of Hygiene and Medical Microbiology Medical University of Innsbruck Innsbruck Austria; ^3^ Servizio di Immunoematologia e Trasfusionale Ospedale S. Orsola‐Malpighi Bologna Italy; ^4^ Istituto per la Sintesi Organica e la Fotoreattività Consiglio Nazionale delle Ricerche Bologna Italy; ^5^ Division of Clinical Biochemistry, Biocentre Medical University of Innsbruck Innsbruck Austria

## Abstract

Hemolytic uremic syndrome (eHUS) is a severe complication of human infections with Shiga toxins (Stxs)‐producing Escherichia coli. A key step in the pathogenesis of eHUS is the interaction of Stxs with blood components before the targeting of renal endothelial cells. Here, we show that a single proteolytic cleavage in the Stx2a A‐subunit, resulting into two fragments (A1 and A2) linked by a disulfide bridge (cleaved Stx2a), dictates different binding abilities. Uncleaved Stx2a was confirmed to bind to human neutrophils and to trigger leukocyte/platelet aggregate formation, whereas cleaved Stx2a was ineffective. Conversely, binding of complement factor H was confirmed for cleaved Stx2a and not for uncleaved Stx2a. It is worth noting that uncleaved and cleaved Stx2a showed no differences in cytotoxicity for Vero cells or Raji cells, structural conformation, and contaminating endotoxin. These results have been obtained by comparing two Stx2a batches, purified in different laboratories by using different protocols, termed Stx2a(cl; cleaved toxin, Innsbruck) and Stx2a(uncl; uncleaved toxin, Bologna). Stx2a(uncl) behaved as Stx2a(cl) after mild trypsin treatment. In this light, previous controversial results obtained with purified Stx2a has to be critically re‐evaluated; furthermore, characterisation of the structure of circulating Stx2a is mandatory to understand eHUS‐pathogenesis and to develop therapeutic approaches.

## 1 INTRODUCTION

Infections with enterohemorrhagic Escherichia coli (EHEC) are the major cause of EHEC‐associated hemolytic uremic syndrome (eHUS), the most common reason for acute renal failure in childhood with a mortality rate of 3–5% (Rosales et al., 2012; Tarr, Gordon, & Chandler, 2005). However, only 5–15% of patients infected with EHEC develop eHUS with the characteristic symptom triad of microangiopathic hemolytic anaemia, thrombocytopenia, and acute renal failure (Tarr et al., 2005).

Shiga toxins (Stxs) represent major EHEC virulence factors in eHUS (Karch, Tarr, & Bielaszewska, 2005). Two different types of toxin, namely, Stx1 and Stx2, are known. Within each of the toxin types, several subtypes have been identified and subtype 2a was shown to correlate significantly more with severe illness in humans, such as eHUS (Friedrich et al., 2002; Orth et al., 2007). Stxs, which belong to the family of ribosome inactivating proteins, are AB5 holotoxins that consist of one enzymatically active A moiety (∼32 kDa) non‐covalently associated with a pentameric B‐subunit (∼7.7 kDa per monomer; Paton & Paton, 1998). The pentameric B‐subunit is responsible for binding to the globotriaosyl‐ceramide (Gb3Cer) or Gb4Cer cellular receptors (Bauwens et al., 2013), mainly expressed by human renal cells such as glomerular endothelial cells (Obrig, 2010).

After internalisation, Stx is transported from early/recycling endosomes to the Golgi apparatus (Garred, van Deurs, & Sandvig, 1995; Mallard et al., 1998) and then to the endoplasmatic reticulum (ER; Sandvig et al., 1992). During the transport, the A‐subunit is cleaved by the protease furin resulting in two fragments, A1 and A2 (∼27.5 kDa and ∼4.5 kDa, respectively). The A1‐fragment remains linked to the A2‐fragment by a disulfide bond between C241 and C260 (Garred et al., 1995) and A2 interacts with the pentameric ring formed by the B subunits. The loop between the two cysteines contains the sequence (Arg‐X‐X‐Arg), the consensus motif for cleavage by furin, which is also recognised by trypsin (Garred et al., 1995). In the ER, the A1 subunit is released from the A2‐B5 complex by reduction of the disulfide bond. The A1 subunit is translocated from the ER into the cytosol or into the nucleus where it inhibits protein synthesis and induces the formation of apurinic sites in chromatin (Bergan, Dyve Lingelem, Simm, Skotland, & Sandvig, 2012; Brigotti et al., 2002; Sandvig & van Deurs, 2005; A. Suzuki et al., 2000).

Before targeting cells in the kidney, Stx binds to blood components, but it is not clear whether the A subunit is still intact then or already cleaved by bacterial or host proteases, and such interactions are involved in the pathogenesis of eHUS. In particular, Stxs are binding to blood cells, like neutrophils, monocytes, or platelets (Brigotti et al., 2008; Brigotti et al., 2013; Cooling, Walker, Gille, & Koerner, 1998; Karpman et al., 2001), and subsequent activation has been reported. As a consequence, formation of leukocyte‐platelet aggregates occurs, followed by the release of 1‐μm‐microvesicles bearing Stxs and other factors such as tissue factor and activated complement factors (Stahl, Sartz, & Karpman, 2011; Stahl, Sartz, Nelsson, Bekassy, & Karpman, 2009). Furthermore, Stx has been demonstrated to activate complement in vitro and to bind factor H (FH; Orth et al., 2009).

Here, we show that, depending on the presence or absence of a single proteolytic cleavage in the disulfide bridged loop of the A subunit, Stx2a has different binding properties for key circulating components involved in the pathogenesis of HUS (neutrophils, FH) and different biological properties (formation of leukocyte‐platelet aggregates). These results have been obtained by comparing two different Stx2a batches purified in two laboratories by using different protocols. This is in line with the previously reported controversial results regarding the effects of purified Stxs.

## 2 RESULTS

### 2.1 Characterisation of Stx2a

Because preliminary data from the Innsbruck laboratory did not confirm Stx2a binding to human neutrophils as previously demonstrated in the Bologna laboratory (Brigotti et al., 2013), we performed a comparative analysis of the biological activity and of the physicochemical properties of the different toxin preparations purified in Innsbruck (Austria) and in Bologna (Italy).

Sequencing analysis confirmed that for both preparations, the *stx2a* genes were identical for the A subunits (Figure S1). The B subunits showed one mismatch at position 36 (Figure S1), which, however, did not change the protein sequence.

The amount of lipopolysaccharide (LPS) in the Stx2a preparations (45 Eu/ml, 56 Eu/mg, Innsbruck; 35 Eu/ml, 67 EU/mg of protein, Bologna) measured by the Limulus assay was comparable.

**Figure 1:**
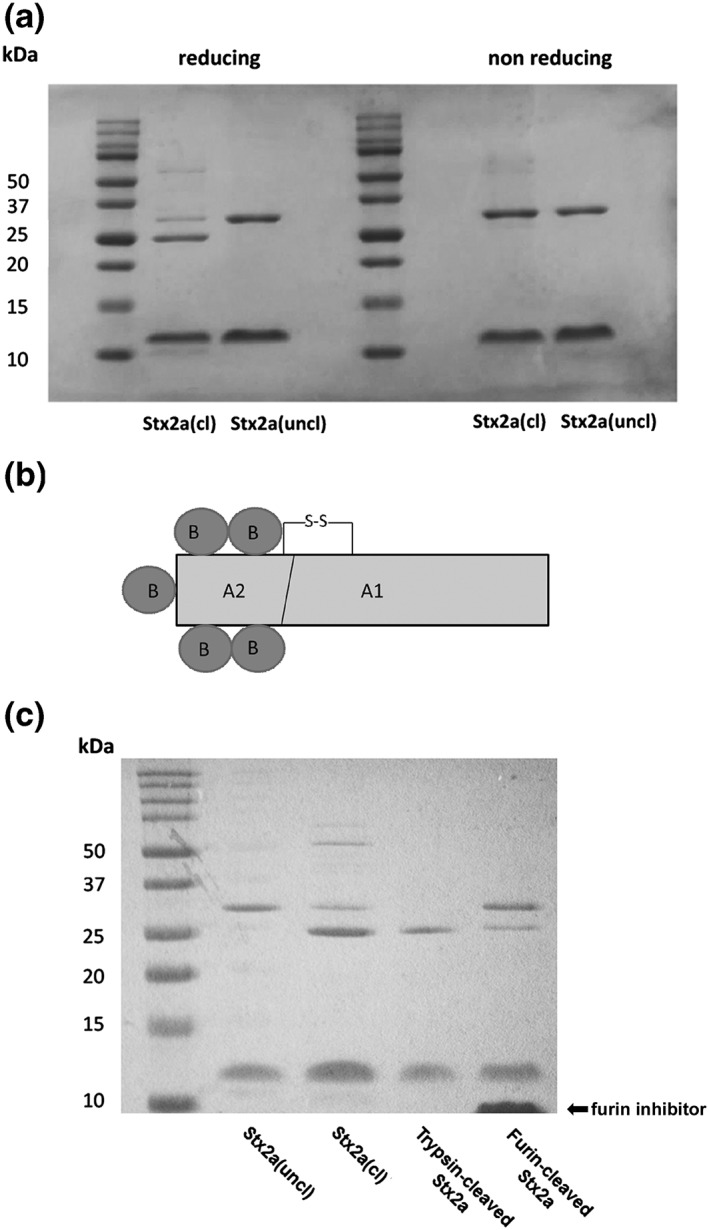
SDS‐PAGE analysis of Stx2a. (a) SDS‐PAGE analysis of Stx2a(cl; 4.5 μg, purified in Innsbruck) and Stx2a(uncl; 4.5 μg, purified in Bologna) performed in non‐reducing and reducing conditions. Molecular mass markers were 10–50 kDa. After electrophoresis, gels were stained with Coomassie blue. The mobility (Rf, distance of protein migration/distance of dye migration) of calibration proteins was plotted versus log of their kDa. The Rf of A subunit and A1 fragment of each preparation allowed calculation of the molecular masses reported under Results. (b) Structure of Stx2a, the trypsin−/furin‐sensitive site in the disulfide bridged loop is indicated. (c) SDS‐PAGE analysis under reducing conditions of Stx2a(cl; 4 μg) and Stx2a(uncl; 4 μg), trypsin‐treated Stx2a, and furin‐treated Stx2a. Gel staining and mobility calculations were performed as described above. Hexa‐D‐arginine is the furin inhibitor indicated in the panel. Densitometric analysis of different SDS‐PAGE gels with which different trypsin and furin‐cleaved Stx2a preparations were resolved showed 85 ± 5% or 53 ± 20% (*n* = 3) A1 fragment, respectively

SDS‐PAGE analysis performed at non‐reducing conditions revealed the presence of two Coomassie‐stained bands corresponding to A and B subunits in Stx2a prepared in Innsbruck (Figure 1a), whereas an additional band appeared at reducing conditions (Figure 1a) having the apparent molecular mass of the A1 fragment (27.6 kDa) and migrating faster than the A subunit (31.7 kDa).

Densitometric analysis showed that Stx2a purified in Innsbruck consists of 16% A chain and of 84% A1 fragment. Because the A1 fragment was observed only in the presence of mercaptoethanol as reducing agent (Figure 1a), we conclude that the A subunit is cleaved in the loop between C241 and C260 and that the two resulting fragments A1 and A2 are linked by an intact disulfide bond (Figure 1b). The cleaved Stx2a from Innsbruck was named Stx2a(cl) throughout the paper. Conversely, Stx2a prepared in Bologna and run at reducing and non‐reducing conditions (Figure 1a) showed a single 31.7 kDa band for the intact A subunit and, hence, was named Stx2a(uncl). Treatment of Stx2a(uncl) with furin, the cell protease involved in the intracellular activation of Stxs (Garred et al., 1995), or with trypsin (Donohue‐Rolfe, Jacewicz, & Keusch, 1988), which mimics the single proteolytic cleavage of the Stx A subunit occurring in cells, reproduced the pattern observed in Stx2a(cl) obtaining the A1‐fragment (27 kDa; Figure 1c).

### 2.2 Cleaved and uncleaved Stx2a differ in the ability to bind human neutrophils

**Figure 2:**
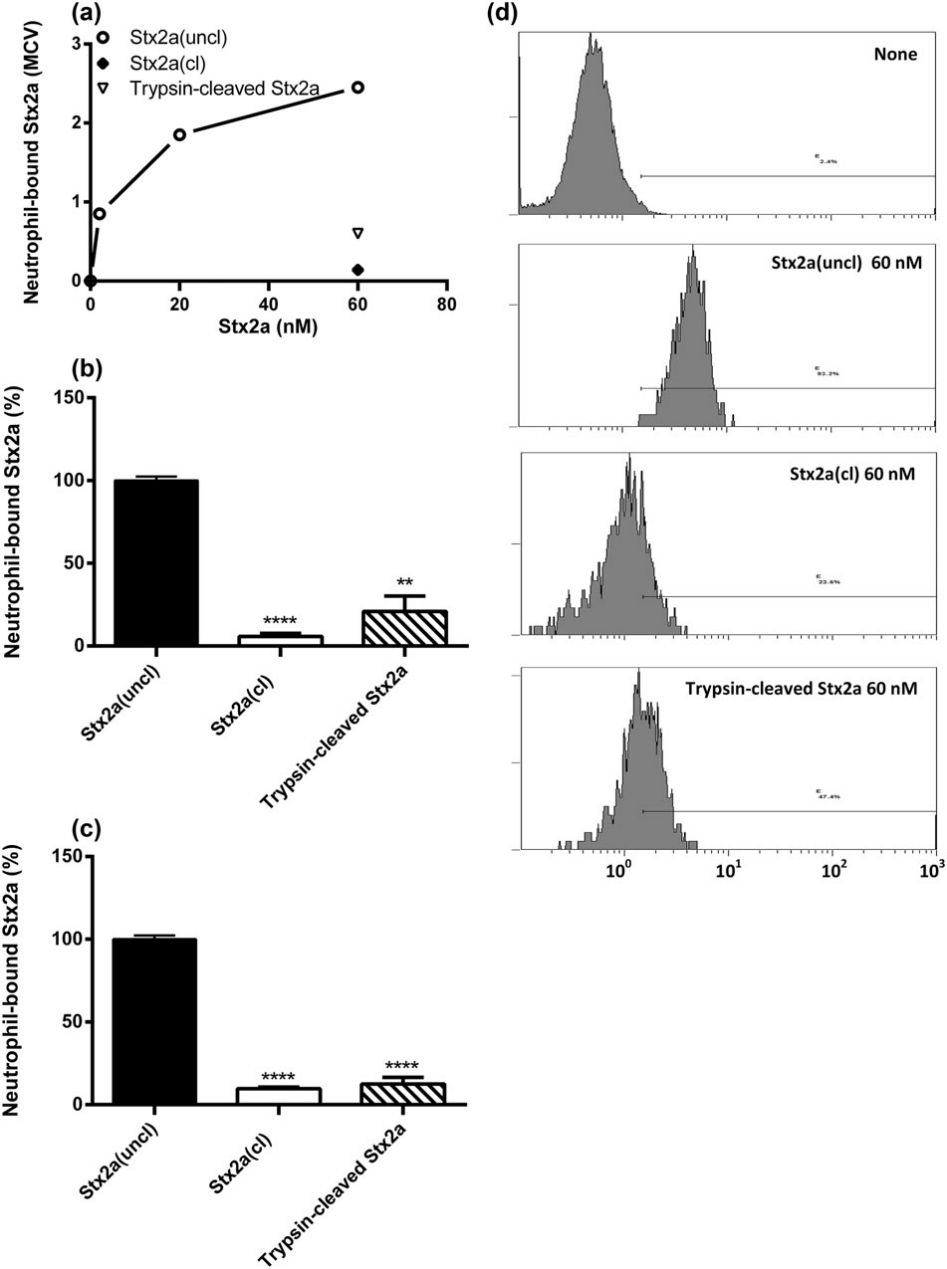
Human neutrophils do not recognise Stx2a(cl) or trypsin‐cleaved Stx2a. (a) Representative experiment, human neutrophils were treated with increasing concentrations of Stx2a(uncl; 2–60 nM) or with Stx2a(cl; 60 nM) and trypsin‐cleaved Stx2a (60 nM), then the binding of the toxins to granulocytes (expressed as MCV) was assessed by indirect flow cytometric analysis. Percentage of Stx2a(cl), Stx2a(uncl), and trypsin‐cleaved Stx2a bound to neutrophils (mean ± S.D.) obtained in three different neutrophil preparations (b) or in human blood samples containing HuSAP (c) challenged with 60‐nM toxins. Significant differences (****P < 0.0001; **P < 0.01) have been obtained with respect to Stx2a(uncl). Under these conditions, the MCVs of isolated neutrophils or neutrophils in blood samples treated with Stx2a(uncl) was 3.13 ± 1.02 or 1.03 ± 0.04 (means ± S.D.; *n* = 3), respectively; these values were used as control to calculate the binding percentages. (d) Representative single histogram analysis showing differential neutrophil‐binding activities of Stx2a(cl), Stx2a(uncl), and trypsin‐cleaved Stx2a. The presence in the binding assay of the complex trypsin/trypsin inhibitor (PMSF) did not affect the binding activity of uncleaved Stx2a(uncl). Cleaved or uncleaved Stx2a were similarly recognised by the B chain‐specific monoclonal antibody to Stx2a used to perform the indirect flow cytometric analysis (Figure S3)

Stx2a(uncl) was found to bind human neutrophils by indirect flow cytometric analysis (Figure 2), giving full saturation of receptors at 60‐nM concentration (Figure 2a), whereas Stx2a(cl) and trypsin‐cleaved Stx2a were not recognised by human neutrophils (Figure 2b,d). This was confirmed with neutrophils from three different healthy donors (Figure 2b). Consistently, furin treatment of Stx2a impaired neutrophil binding to extents (60.8 ± 8.6% inhibition with respect to untreated Stx2a(uncl), *n* = 3, *P* = 0.0066) related to the amount of generated A1 fragment (47%).

To achieve a more physiological situation, binding of Stx2a to neutrophils was also investigated in human blood. Binding of cleaved or uncleaved toxins to neutrophils was not influenced by the presence of blood components (Figure 2c).

### 2.3 Cleaved and uncleaved Stx2a differ in the ability to form leukocyte/platelet aggregates in human blood

**Figure 3:**
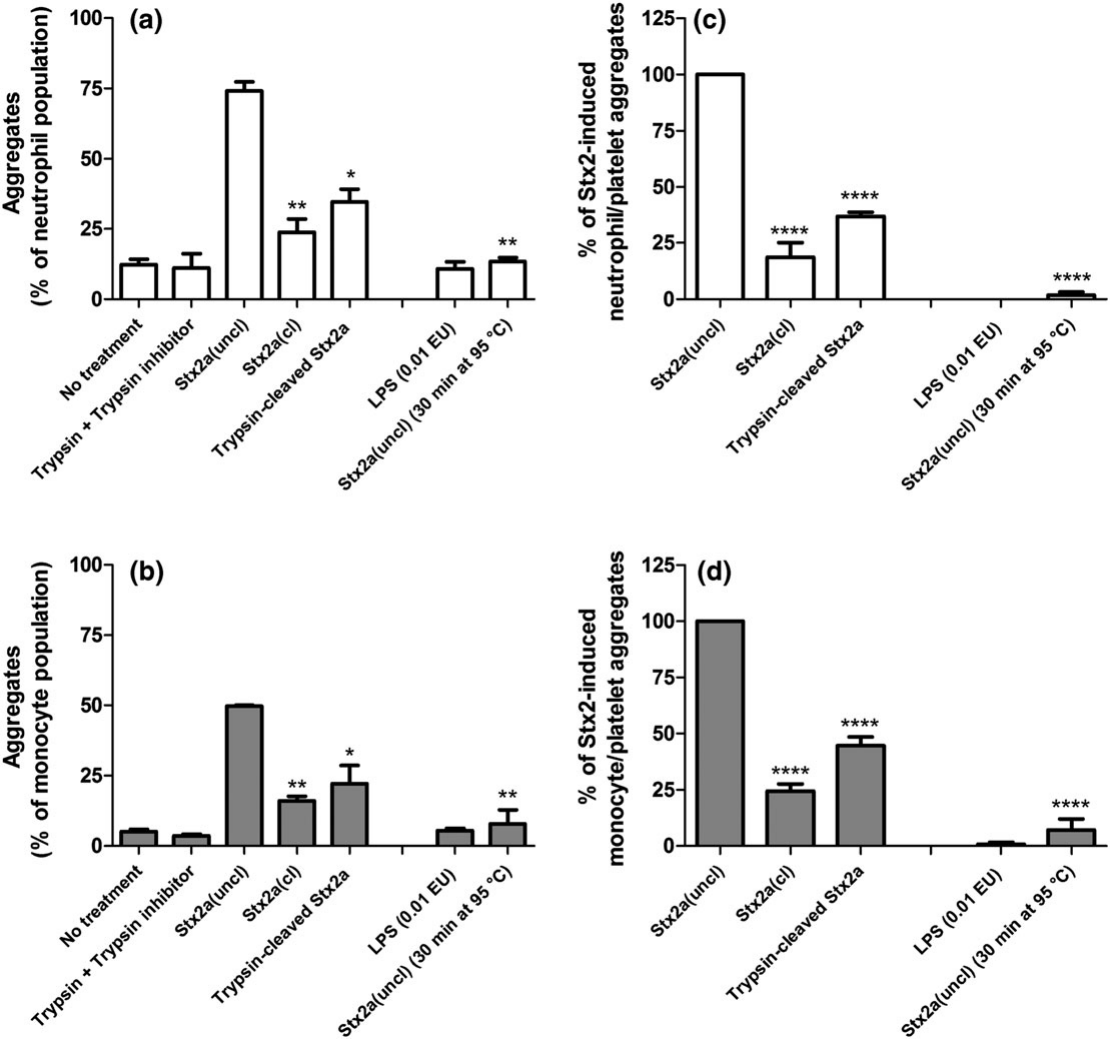
Stx2a(cl) or trypsin‐cleaved Stx2a added to human blood are not efficient in triggering the formation of leukocyte/platelet aggregates. Whole human blood was treated with 1 nM Stx2a(cl), Stx2a(uncl), or trypsin‐cleaved Stx2a. When indicated lipopolysaccharide amount (0.01 EU) similar to that contaminating Stx2a preparations was added in the assay. Then, the formation of neutrophil/platelet or monocyte/platelet aggregates was assessed by direct flow cytometric analysis. Values obtained in a representative experiment expressed as percentage of aggregates on the neutrophil (a) or monocyte (b) populations; data are means ± SD (*n* = 2). (c, d) Values minus the appropriate controls (no treatment or trypsin + trypsin inhibitor) from three independent experiments were used to calculate the percentage of total aggregates with respect to Stx2a(uncl); data are means ± SD (*n* = 3). After incubation of human blood with Stx2a(uncl), the percentages of neutrophil/platelet aggregates and of monocyte/platelet aggregates on the whole population of neutrophils or monocytes were 55.0 ± 8.0% and 47.5 ± 14.2% (mean ± S.D., n = 3), respectively. *P < 0.05, **P < 0.01, ****P < 0.0001 with respect to Stx2a(uncl) are indicated in the figure

Although Stx2a(uncl) was fully active with respect to leukocyte/platelet aggregates formation, Stx2a(cl) and trypsin‐cleaved Stx2a did induce lower percentages of leukocyte/platelet aggregates both in representative experiments (Figure 3a,b) and in confirmation with three healthy donors (Figure 3c,d). Addition of LPS amounts present in Stx2a preparations to blood produced no aggregates, as well as the addition of heat‐treated Stx2a(uncl).

### 2.4 Cleaved and uncleaved Stx2a differ in the ability to interact with complement factor H

**Figure 4:**
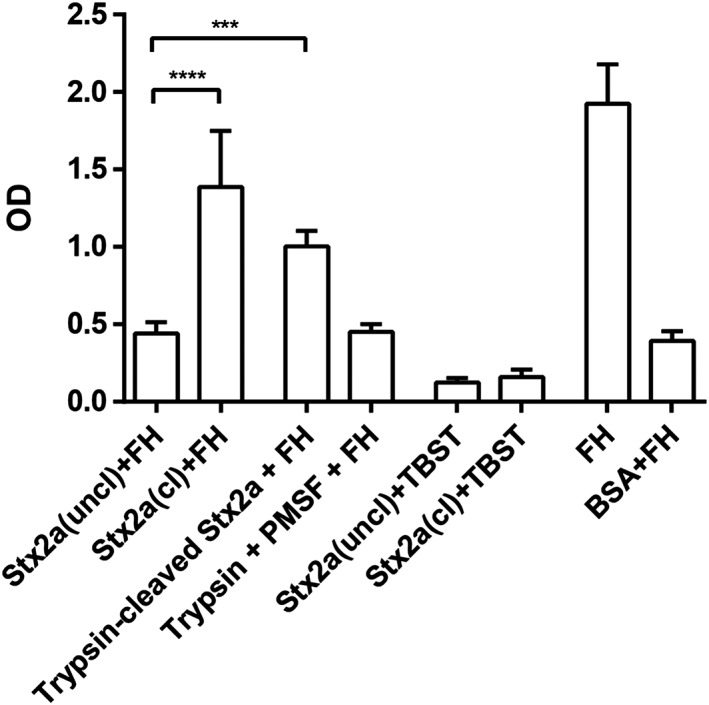
Stx2a(cl) and trypsin‐cleaved Stx2a binds to complement FH. Immobilised Stx2a(cl) or Stx2a(uncl) or trypsin‐cleaved Stx2a (2 μg/well each) was incubated with FH (2 μg). As controls trypsin + trypsin inhibitor (PMSF) or BSA were incubated with FH. Additional controls were performed by incubating the proteins with buffer (TBST) instead of FH. As positive control FH was used. The binding capacity of Stx2a(cl) and trypsin‐cleaved Stx2a to FH were significantly higher compared with Stx2a(uncl) and all negative controls (*P* < 0.001 for all). ***P < 0.001, ****P < 0.0001

Binding of Stx2a to complement factor H, assessed by ELISA, was confirmed for Stx2a(cl) and was also detected for trypsin‐cleaved Stx2a but not for Stx2a(uncl; *P* < 0.001 for all; Figure 4). FH binding was also evaluated with LPS (amount present in Stx2a preparations) and was found to have no effect (data not shown). Thus, the binding patterns of the different toxin forms to FH was opposite (cleaved Stx2a active, uncleaved Stx2a inactive) with respect to aggregate formation or interaction with granulocytes (cleaved Stx2a inactive, uncleaved Stx2a active).

### 2.5 Cleaved and uncleaved Stx2a do not activate human platelets

It has been previously reported that Stx can activate platelets (Karpman et al., 2001), hence, concurring to the development of thrombocytopenia during eHUS. Both Stx2a(uncl) and Stx2a(cl) were unable to activate platelets, which conversely are sensitive to thrombin used as positive control (Figure S2) as assessed by increased surface expression of CD62P.

### 2.6 Cleaved and uncleaved Stx2a are fully active toxins for Gb3Cer‐expressing cells

**Figure 5:**
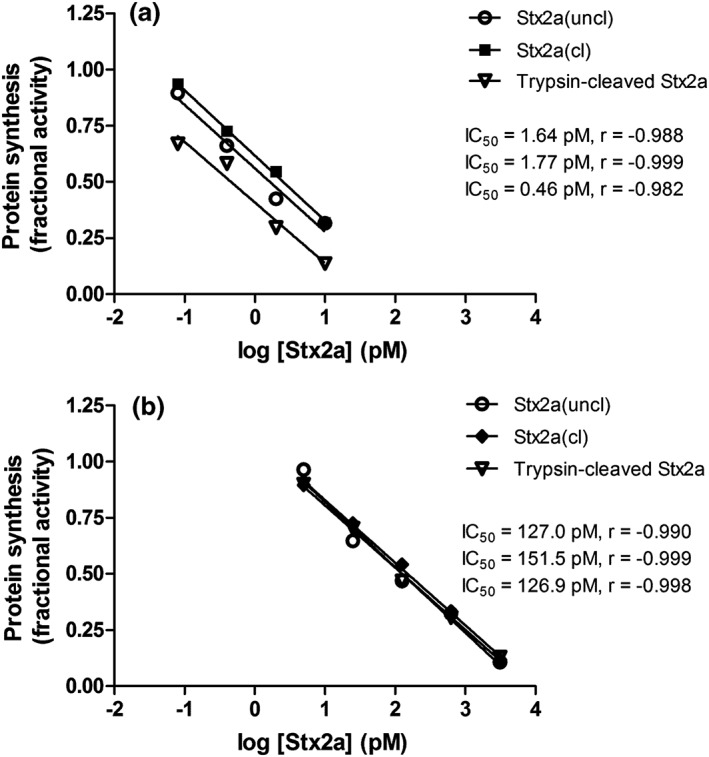
Inhibition of protein synthesis in Raji cells by Stx2a(cl), Stx2a(uncl), and trypsin‐cleaved Stx2a. Raji cells were treated 3 hr with different concentrations of toxins in the absence (a) or in the presence (b) of 10% human serum then protein synthesis was measured in the presence of labelled leucine. IC_50_ was calculated by the linear regression between fractional activity and the log of Stx2a concentrations

**Figure 6:**
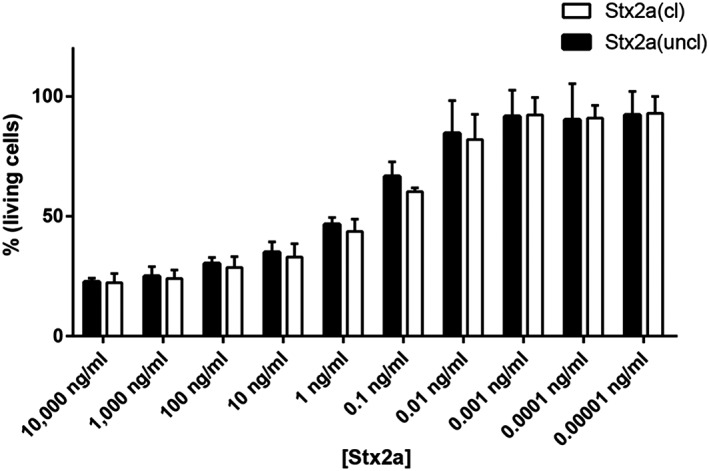
Cytotoxicity profiles of Stx2a(cl) and Stx2a(uncl) on Vero cells are very similar. Vero cells were incubated 48 hr with a broad range (10 fg/ml‐10 μg/ml) of Stx2a concentrations (150 aM‐150 nM). Residual cell density was measured by crystal violet method, and the percentage of living cells is shown on the y‐axis

Although differing in neutrophil binding, aggregate formation and FH‐binding abilities, the different toxin forms were found to be fully active and similar in inhibiting translation in sensitive cells endowed with the receptor Gb3Cer such as Raji cells (Figure 5a). After a short incubation with Stx2a, translation was determined in the presence of a radioactive amino acid as previously described (Arfilli et al., 2015). The calculated IC_50_ were very similar and as expected for fully active Stxs (Brigotti et al., 2002; Brigotti et al., 2007; Brigotti et al., 2011). It has been shown that the presence of human serum strongly reduced the inhibitory power of Stx2a on this system because of the presence of HuSAP (Arfilli et al., 2015). This circulating protein complex, by capturing Stx2a, prevents the targeting of Gb3Cer‐containing cells by the toxin (Armstrong et al., 2006; Kimura, Tani, Matsumoto Yi, & Takeda, 2001; Marcato, Vander Helm, Mulvey, & Armstrong, 2003). When adding human serum, translation inhibition was similarly strongly impaired for cleaved or uncleaved toxins (Figure 5b). The cytotoxicity profiles on Vero cells, chosen to reveal long‐term cytopathic effects, were also found to be similar (Figure 6).

### 2.7 Cleaved and uncleaved Stx2a do not differ in conformation

**Figure 7:**
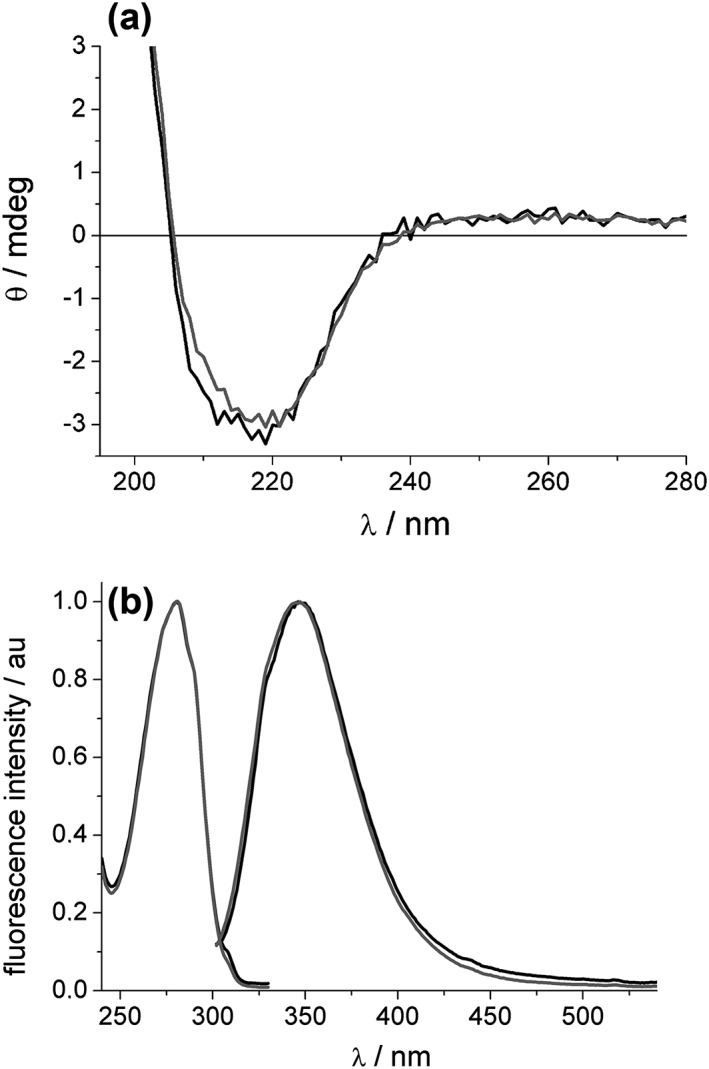
Conformation and intrinsic fluorescence of Stx2a. (a) Ellipticity of 1 μM Stx2a(uncl; black) and Stx2a(cl; grey) in PBS at 22°C, 0.1 cm cell, examined by circular dichroism. (b) Fluorescence spectra (right, excitation at 295 nm) and excitation spectra (left, emission at 345 nm) of 1 μM Stx2a(uncl; black) and Stx2a(cl; grey) in PBS at 22°C

To gain information on the effect of cleavage on the conformation of the toxins, Stx2a(cl) and Stx2a(uncl) were examined by circular dichroism (CD) and fluorescence. The CD spectra of the two proteins were almost identical indicating conformational similarity (Figure 7a). The spectrum exhibits a negative minimum at 220 nm and a less intense slightly positive band above 235 nm. According to the CD spectral profiles of proteins of known structure (Brahms & Brahms, 1980), we can conclude that both contain a significant fraction of α‐helix structure and only a small fraction of β‐sheet structures.

This was further confirmed by their fluorescence behaviour (Figure 7b). Stx2 possesses 12 tryptophan (Trp) residues, the dominant fluorophores in proteins, two of them in the A subunit and two in each of the five B subunits. The fluorescence spectra of the two proteins were very similar in shape and peak at 347 nm, a quite red shifted value indicative of an average polar environment for Trp residues in both proteins. The maximum in the excitation spectra at 281, as well as the shape, corresponds to Trp absorption in polar environment. Global analysis of the decay profiles for each protein evidenced two lifetime (τ_i_) components that were almost the same for the two proteins (Stx2a(cl): τ_1_ = 2.64 ns, τ_2_ = 7.22 ns; Stx2a(uncl): τ_1_ = 2.50 ns, τ_2_ = 7.33 ns) and have similar fractional contributions. The presence of two lifetime components, similar in both protein samples, points to the presence of at least two types of Trp environments of different average polarity.

The photophysical study allowed to conclude that no significant conformational changes occurred in the two proteins in spite of the differences observed in their structure.

## 3 DISCUSSION

Stxs are believed to be the main virulence factors in eHUS, and amongst the Stx subtypes, Stx2a is strongly associated with severe disease, including eHUS (Orth et al., 2007). Thus, several in vitro studies have been performed with purified Stx2a to elucidate the pathomechanism of this most extreme outcome of EHEC infection. However, for Stx purification, there is no standardised protocol available and, thus, different and often poorly detailed Stx purification methods, which employ different Stx‐producing strains have been used.

Different capabilities of Stx2a have been described that might contribute to the pathogenesis of eHUS. These characteristics include cytotoxicity against various cells, often using Vero cells as model, protein synthesis inhibition, neutrophil binding, platelet binding and activation, formation of leukocyte/platelet aggregates, binding to different serum proteins, such as HuSAP or complement factor H, and complement activation (Bauwens et al., 2011; Brigotti et al., 2013; Brigotti et al., 2008; Cooling et al., 1998; Karpman et al., 2001; Lentz, Leyva‐Illades, Lee, Cherla, & Tesh, 2011; Marcato et al., 2003; Orth et al., 2009; K. Poolpol et al., 2014). However, several of these Stx2a‐induced features have been controversially discussed. Either the Stx2a‐induced effect was not comparable in magnitude, or some of these effects could not even be reproduced by other groups.

Investigation of Stx2a‐induced capabilities of Stx2a(uncl) or of cleaved forms (Stx2a(cl) and trypsin‐cleaved Stx2a) revealed comparable results regarding cytotoxic activity and absent activation of platelets for both preparations. Conversely, in the case of key Stx2a‐induced capabilities, striking differences were found: Stx2a(uncl) was confirmed to bind to human neutrophils and to trigger leukocyte/platelet aggregate formation whereas cleaved Stx2a, such as Stx2a(cl) and trypsin‐cleaved Stx2a, was ineffective. In contrast, binding to FH was confirmed for Stx2a(cl) and was also detected for trypsin‐cleaved Stx2a; Stx2a(uncl) failed to bind FH.

Limulus assay revealed no significant differences in the low‐LPS amount that contaminated Stx preparations. Moreover, running experiments (neutrophil binding, leukocyte‐platelet aggregate formation, and FH binding) with these LPS amount did not show any effect.

Structure analyses of the Stx2a preparations revealed conformational similarity of both toxins including a comparable content of α‐helices and β‐sheet structures. However, SDS‐PAGE analyses showed that Stx2a(cl) A subunit is cleaved in two fragments whose molecular masses correspond to A1 and A2 fragments. The A fragments of Stx2a(cl) are linked by a disulfide bond, shown by the fact that only the mercaptoethanol‐reduced gels resolved A1 and A2 fragments, whereas use of non‐reducing sample buffer revealed only one band for the A subunit. Cleavage of the A subunit was found to happen early in the purification procedure of Stx2a(cl) at the sonication step (data not shown); this is in agreement with the fact that this step is lacking in the purification method yielding the Stx2a(uncl). The structural change was reproduced by treating Stx2a(uncl) with trypsin and furin to mimic the cleavage observed during the Stx2a(cl) purification.

Stx2a has been described to bind to human neutrophils via its A subunit at TLR4 (Brigotti et al., 2013). The fact that Stx2a(cl) and trypsin‐cleaved Stx2a did not interact with human granulocytes suggests that the binding site for neutrophils might be located close to the cleaved area of the A subunit but not in the active site of Stx2a, as cytotoxic activity and protein synthesis were unchanged. Furthermore, the fact that Stx2a(uncl) induced a significantly higher percentage of neutrophil and monocyte‐platelet aggregate formation than Stx2a(cl) might indicate an important role of TLR4 in these phenomena. It should be noted that all the actors involved (platelets, monocytes, and neutrophils) express this receptor.The Stx2a B subunit has previously been suggested to contain the binding site for FH (Poolpol, 2012). We hypothesize that nicking of the A subunit might influence the quaternary structures of the whole toxin affecting A/B‐subunit interactions, thus explaining the different abilities of Stx2a preparations concerning the binding to FH. However, it seems worth to note that cytotoxic activity of both toxin preparations was comparable indicating that the receptor binding sites located in the B subunit (Bauwens et al., 2013) of Stx2a were not affected and that also the cooperation between B and A chains (binding to Gb3Cer and intracellular enzymatic action, respectively) was operative for both toxin batches.

Cleavage of the Stx2a A subunit has been described during the cellular transport of Stx2a; the A1 fragment is released in the ER and subsequently conveys protein synthesis inhibition at the ribosome (Sandvig et al., 1992). However, nicking of the A subunit might also occur in the intestinal mucus (Melton‐Celsa, Darnell, & O'Brien, 1996) or is likely induced by bacterial proteases released by lysed EHEC during infection. In this case, one can assume the presence of different proportion of cleaved and uncleaved Stx2a in blood during the natural course of EHEC infections and in the transition from hemorrhagic colitis to eHUS. There are very limited data showing presence of Stx2a in blood of EHEC‐infected patients. Lopez and co‐workers have detected free Stx2a in sera of three EHEC‐infected patients during the prodromal intestinal phase before the onset of eHUS (Lopez et al., 2012), and He and co‐authors have found only very low amounts of Stx2a in sera of eight patients with overt eHUS (He et al., 2015). Arfilli and co‐workers have demonstrated presence of functional active Stx2a in sera of three EHEC‐infected patients during hemorrhagic colitis (Arfilli et al., 2015). However, none of these studies presented any data on the structure and/or conformation of Stxs in the blood of eHUS patients. Thus, it is not clear at which step of eHUS pathogenesis Stx2a cleavage could occur and to which extent.

Furthermore, it cannot be stated whether and to what extent neutrophil binding, leukocyte‐platelet aggregation, and factor H binding occur and contribute to eHUS pathogenesis. Interestingly, uncleaved and cleaved Stx2a seem to participate similarly in HuSAP‐related effects as supported by our experiments in the presence of human blood and serum. This blood protein prevents the targeting of Gb3Cer‐expressing cells by interacting with Stx2a and, at the same time, promotes the interaction of the toxin with human neutrophils and subsequent steps involved in the pathogenesis of eHUS.

We actually speculate that for the binding to neutrophils, it is mandatory that the toxin remains uncleaved because these cells do not express Gb3Cer. Then, during the subsequent pathogenetic steps (formation of aggregates and release of microvesicles) or in the vicinity of the target organ, the toxin could get cleaved, allowing the interaction with factor H. Moreover, circulating cells co‐expressing TLR4 and Gb3Cer, such as monocytes and platelets, could interact with cleaved (through Gb3Cer) or uncleaved (through TLR4 and Gb3Cer) Stx2a. Hence, the relative proportion of cleaved and uncleaved Stx2a on these cells could determine (a) different amounts of the deriving aggregates and microvesicles and (b) different pathogenic factor contents (activated complement components) in aggregates and microvesicles. Finally, cleavage of cell surface‐associated Stx2a could represent a bound/unbound mechanism allowing toxin delivery to target endothelia after transport by TLR4‐expressing circulating cells.

In conclusion, this study reveals more insights into (a) the effects of purification protocols on the structure and function of isolated Stx2a (similar considerations may also be applicable to other toxins and proteins in general) and (b) the structure‐related mechanisms underlying key points of eHUS pathogenesis such as Stx2a‐binding to neutrophils, leukocyte‐platelet aggregate formation, and FH binding abilities.

Different Stx purifications utilising different bacterial strains and different purification protocols lead to different capabilities of the toxin; thus, there is urgent need for a standardised protocol for Stx purification and a re‐evaluation of previous findings using purified Stx2a. On the other hand, based on the structure/function relationship described here, an important goal would be the characterisation of the structure of Stx2a circulating in EHEC‐infected patients and its relation to the pathogenetic process, that is, development of eHUS or recovery after the intestinal prodromal phase. However, a formal evidence of the presence of the cleaved toxin in patients' circulation during EHEC infections is lacking. Work in progress in our laboratories is aimed at the specific detection of cleaved and uncleaved toxin forms in pathophysiological models of HUS.

Understanding the real structure and conformation of eHUS‐inducing Stx2a would allow development of appropriate animal models aimed at studying the impact of the different toxin form (cleaved or uncleaved) on eHUS pathogenesis. This may improve strategies for the prevention of the life‐threatening transition from hemorrhagic colitis to eHUS in EHEC‐infected children.

## 4 EXPERIMENTAL PROCEDURES

### 4.1 Proteins and antibodies

LPS from E. coli serotype O111B4 was obtained from Alexis Biochemicals (Roma, Italy); FH from Calbiochem (Darmstadt, Germany); bovine serum albumin (BSA), thrombin, trypsin type XI (from bovine pancreas (T1005)), furin (F2677, 2000 U/ml), the furin inhibitor hexa‐D‐arginine (SCP0148), 4‐nitrophenyl phosphate disodium salt hexahydrate, and alkaline phosphatase (AP)‐conjugated anti‐sheep antibody (Ab) from Sigma Aldrich (Taufkirchen, Germany, or Milan, Italy); sheep anti‐human FH (Ab) from Binding Site (Birmingham, UK); mouse anti‐Stx2a Ab (Stx2‐BB12) from Toxin Technology (Sarasota, FL); allophycocyanin (APC)‐conjugated CD42b Ab from BD Biosciences (San Jose, CA); phycoerythrin (PE)‐conjugated CD62P Ab from BioLegend (San Diego, CA); FITC‐labelled anti‐CD16, anti‐CD65, anti‐CD14, goat anti‐mouse IgG, PE‐labelled anti‐CD41, PC5‐labelled anti‐CD16 from Beckman Coulter (Miami, FL). Blood (in 3.2% citrate) for the preparation of platelet‐rich plasma (PRP) or serum were collected from three healthy volunteers after informed consent and with an existing ethical vote.

### 4.2 Toxin purification

E. coli strain R82 (pJES) 120DH5α and E. coli C600 (933 W) were supplied by Mohamed Karmali (Ottawa, Canada) and by Alison O'Brien (Bethesda, MD), respectively. In the former case, Stx2a (named Stx2a(cl) throughout the paper) was purified in Innsbruck (Austria) by the following steps: culture, sonication, concentration on Amicon filters, dialysis, hydroxyapatite chromatography, dialysis, concentration on Amicon filters, chromatofocusing using DEAE Sepharose fast flow ion exchanger (Logan, Lagerlund, & Chamow, 1999), ammonium sulfate precipitation, and dialysis. In the second case, Stx2a (called Stx2a(uncl) throughout the paper) was isolated in Bologna (Italy) as described in a previous study (Matussek et al., 2003): culture, ammonium sulfate precipitation, dialysis, receptor analog affinity chromatography on Galα1‐4Galβ‐*O*‐spacer–BSA–Sepharose 4B (Glycorex, Lund, Sweden), concentration on Amicon filters. After purification, both Stx preparations were passed through ActiCleanEtox column (Sterogene Bioseparations, Carlsbad, CA) to remove trace endotoxin contaminants. The amount of contaminating LPS was determined by using the Endosafe®Endochrome‐K™ assay (Charles River, Charleston, SC). The test method is based on the Chromogenic technique as described elsewhere (United States Pharmacopeial Convention, Bacterial Endotoxins Test). Toxins were analysed by SDS‐PAGE in reducing and non‐reducing conditions using a 16% (wt/vol) gel followed by staining with Coomassie blue.

### 4.3 Cleavage of Stxs with trypsin or furin

Stx2a(uncl) (4 μg) was incubated in 10 μl PBS pH 7.5 for 1 hr at 37°C with 50 ng of trypsin (1 mg/ml in 0.1 mM HCl, diluted to 0.05 ng/ml with PBS). Then, 0.7 ng of the trypsin inhibitor PMSF (1 mg/ml in absolute ethanol diluted to 0.7 μg/ml with water) was added for 10 min at 37°C. Alternatively, Stx2a(uncl; 4 μg) was incubated with 2 U of furin in 10 μl PBS pH 7.5 containing 1 mM CaCl_2_ for 1 hr at 37°C. Then, 4 μl of the furin inhibitor hexa‐D‐arginine 1.05 mM (Cameron, Appel, Houghten, & Lindberg, 2000) was added for 10 min at 37°C.

### 4.4 Sequencing of *stx2a* gene

The *stx2a* subunit A and B genes were amplified using primers and PCR conditions described elsewhere (Orth et al., 2007). Purified PCR products were analysed on the Applied Biosystems 3500 Genetic Analyser according to standard Sanger sequencing procedures and according to the manufacturer's protocol. Sequencing data were processed and analysed with Sequencing 5.2 Analysis Software (Applied Biosystems). Sequences were aligned using Serial Cloner Software Version 2.6.

### 4.5 Isolation of human neutrophils and binding assays with toxins

Neutrophils (99.7% purity) were isolated under endotoxin low conditions from buffy coats of several healthy donors by centrifugation over Ficoll‐Paque, dextran sedimentation, erythrocytic lysis, and negative selection of contaminant cells (Stemcell Technologies, Vancouver, BC, Canada) as in the previous study (Brigotti et al., 2013; Cassatella et al., 1988). For binding experiments with Stx2a, Eppendorf tubes were precoated with PBS containing 1% endotoxin low (≤1 endotoxin unit/mg) BSA to avoid nonspecific loss of the toxins (van Setten, Monnens, Verstraten, van den Heuvel, & van Hinsbergh, 1996). Neutrophils (5 × 10^5^) were incubated 90 min at 37°C with different concentrations of Stx2a (2–60 nM) in 250 μl of the same buffer (PBS‐BSA). After incubation, the cells were collected by centrifugation at 200 × g for 5 min and washed three times. Stx2a bound to neutrophils was detected by flow cytometry using a mouse monoclonal anti‐Stx2a antibody as previously described. Binding of Stx2a to cells was quantified as mean channel value of fluorescence (Tazzari et al., 2004). The presence of the complexes trypsin/PMSF or furin/hexa‐D‐arginine in neutrophil‐binding assays did not affect the binding activity of Stx2a(uncl). Cleaved or uncleaved Stx2a were similarly recognised (Raji assay, see below) by the B chain specific monoclonal antibody (Figure S3).

### 4.6 Neutrophil‐binding assay with Stx2a in human blood

Blood (250 μl) from healthy donors was challenged with Stx2a (60 nM) for 90 min at 37°C. Cells were collected by centrifugation at 2700 × g for 15 min at room temperature. After erythrocytic lysis, washed leukocytes were obtained, and the binding of Stx2a to neutrophils was assessed by indirect flow cytometric analysis as described above.

### 4.7 Detection of leukocyte/platelet aggregates by direct flow cytometry

Whole‐blood samples (1 ml) from various donors were incubated for 4 hr at 37°C with Stx2a (1 nM). After erythrocytic lysis, samples were incubated with PE‐labelled anti‐CD41 to reveal platelets and FITC‐labelled anti‐CD14 for monocyte detection or PC5‐labelled anti‐CD16 to identify neutrophils on gated granular cells. Controls with appropriate isotype Abs were performed. CD14/CD41 double‐positive cell populations or CD16/CD41 granular double‐positive cell populations were identified as monocyte/platelet or neutrophil/platelet aggregates, respectively.

### 4.8 Analysis of Stx2a binding to FH

Binding of Stx2a to FH was detected by ELISA as previously described (Orth et al., 2009). The different Stx2a preparations were immobilised at 2 μg/well each in a microtiter plate followed by addition of FH at 2 μg/well for 6 hr at 37°C.

### 4.9 Platelet activation assay

To evaluate whether Stx2a enhanced platelet activity, PRP was incubated for 90 min at 37°C with various concentrations of purified Stx2a (1–100 ng/ml). For positive control, PRP was treated with thrombin (0.1 U/ml), for negative control with medium only. Platelet activation and subsequent secretion of α‐granules were evaluated via CD62P (P‐selectin) quantification using FACScan apparatus (Becton Dickinson, NJ; Lu & Malinauskas, 2011). The antigen CD42b, present on both resting and activated platelets, was used for platelet identification (H. Suzuki, Yamamoto, Tanoue, & Yamazaki, 1987). Results were given as the mean fluorescence intensity.

### 4.10 Radioactive translation inhibition assay with Raji cells

Inhibition of protein synthesis by Stx2a was assayed as previously described (Arfilli et al., 2015). Briefly, after 3‐hr incubation of Raji cells (0.3 × 10^6^, supplied by Andrea Bolognesi, Bologna, Italy) with toxins (16 fM‐3.125 nM) in the presence or absence of 10% human sera, [^3^H] leucine (2 μCi) was added. Radioactive proteins synthesised during 1‐hr incubation were collected on glass filters after treatment of cell pellets with KOH 0.1 N followed by 10% trichloroacetic acid (TCA) precipitation. After extensive washing with 5% TCA, the radioactivity was measured by a liquid‐scintillation β counter.

### 4.11 Vero cells cytotoxicity assay

The cytotoxicity assay using Vero cells was performed as previously described (Gentry & Dalrymple, 1980; Russo, Melton‐Celsa, Smith, & O'Brien, 2014). Briefly, cells were incubated with toxins (150 aM‐150 nM) for 48 hr. Detection was done via 1% crystal violet staining.

### 4.12 Photophysical measurements

Absorption spectra were recorded on a Perkin Elmer l650 spectrophotometer. CD spectra were obtained with a Jasco J‐715 spectropolarimeter (2 nm resolution) and fluorescence spectra on a Edinburgh spectrofluorimeter with right angle detection geometry at 22°C. A time‐correlated single photon counting system (IBH Consultants Ltd.) was used to study the time‐resolved fluorescence of air‐equilibrated solutions, excited at 278 nm with a Nano‐LED source. Decay profiles were collected at 330 nm and analysed using a multiexponential function as described (Brigotti et al., 2011; Lakowicz, 2006).

### 4.13 Statistical analyses

Statistical analysis was performed with GraphPad Prism 5. Differences in continuous variables were tested with a *t* test after controlling the normality of their distribution. A *P* value <0.05 was considered statistically significant. Correlation between variables was assessed using a Pearson correlation coefficient.

## Supporting information


**FIGURE S1** Sequence of the genes encoding the A and the B subunits of Stx2a purified in Innsbruck (E. coli strain R82 (pJES) 120DH5α) and in Bologna (E. coli C600 (933 W).
**FIGURE S2** Lack of P‐selectin (CD62P) expression as a measure of platelet activation in PRP in the presence of Stx2a(cl) and Stx2a(uncl). The vertical axis represents the mean fluorescence intensity. FACS analysis of platelet activation was performed by incubation of PRP with medium, thrombin (positive control) and various concentrations of Stx2a(cl) or Stx2a(uncl). The results are the means ± S.D. of five separate experiments.
**FIGURE S3** Effect of monoclonal antibody to Stx2a on the inhibition of Raji cells protein synthesis by Stx2a(uncl) and trypsin‐cleaved Stx2a. Raji cells were treated 3 h with 2.5 pM toxins in the absence or in the presence of the monoclonal antibody (10 μg) used to detect Stx2a bound to neutrophils (Stx2‐BB12) then protein synthesis was measured in the presence of labelled leucine as described under Experimental procedures. *** *p* < .001
