# Supplementary material for: The structure of the Shiga toxin 2a A‐subunit dictates the interactions of the toxin with blood components
Source: Cell Microbiol. 2019 Jan 18;21(5):e13000. doi: 10.1111/cmi.13000 (PMC6492301; doi:10.1111/cmi.13000)
Supplement: Supplementary file 1 — FIGURE S1 Sequence of the genes encoding the A and the B subunits of Stx2a purified in Innsbruck (E. coli strain R82 (pJES) 120DH5α) and in Bologna (E. coli C600 (933 W). FIGURE S2 Lack of P‐selectin (CD62P) expression as a measure of platelet activation in PRP in the presence of Stx2a(cl) and Stx2a(uncl). The vertical axis represents the mean fluorescence intensity. FACS analysis of platelet activation was performed by incubation of PRP with medium, thrombin (positive control) and various concentrations of Stx2a(cl) or Stx2a(uncl). The results are the means ± S.D. of five separate experiments. FIGURE S3 Effect of monoclonal antibody to Stx2a on the inhibition of Raji cells protein synthesis by Stx2a(uncl) and trypsin‐cleaved Stx2a. Raji cells were treated 3 h with 2.5 pM toxins in the absence or in the presence of the monoclonal antibody (10 μg) used to detect Stx2a bound to neutrophils (Stx2‐BB12) then protein synthesis was measured in the presence of labelled leucine as described under Experimental procedures. *** p < .001 [file CMI-21-na-s001.docx]

Seq_1: gene encoding the A chain of the Stx2a purified in Bologna (Italy)

Seq_2: gene encoding the A chain of the Stx2a purified in Innsbruck (Austria)

Similarity: 528/528 (100.00 %)

Seq_1 1 ATTCTCTCTGTATCTGCCTGAAGCGTAAGGCTTCTGCTGTGACAGTGACAAAACGCAGAA 60

||||||||||||||||||||||||||||||||||||||||||||||||||||||||||||

Seq_2 1 ATTCTCTCTGTATCTGCCTGAAGCGTAAGGCTTCTGCTGTGACAGTGACAAAACGCAGAA 60

Seq_1 61 CTGCTCTGGATGCATCTCTGGTCATTGTATTACCACTGAACTCCATTAACGCCAGATATG 120

||||||||||||||||||||||||||||||||||||||||||||||||||||||||||||

Seq_2 61 CTGCTCTGGATGCATCTCTGGTCATTGTATTACCACTGAACTCCATTAACGCCAGATATG 120

Seq_1 121 ATGAAACCAGTGAGTGACGACTGATTTGCATTCCGGAACGTTCCAGCGCTGCGACACGTT 180

||||||||||||||||||||||||||||||||||||||||||||||||||||||||||||

Seq_2 121 ATGAAACCAGTGAGTGACGACTGATTTGCATTCCGGAACGTTCCAGCGCTGCGACACGTT 180

Seq_1 181 GCAGAGTGGTATAACTGCTGTCCGTTGTCATGGAAACCGTTGTCACACCGGGCACTGATA 240

||||||||||||||||||||||||||||||||||||||||||||||||||||||||||||

Seq_2 181 GCAGAGTGGTATAACTGCTGTCCGTTGTCATGGAAACCGTTGTCACACCGGGCACTGATA 240

Seq_1 241 TATGTGTAAAATCTGAAAAACGGTAGAAAGTATTTGTTGCCGTATTAACGAACCCGGCCA 300

||||||||||||||||||||||||||||||||||||||||||||||||||||||||||||

Seq_2 241 TATGTGTAAAATCTGAAAAACGGTAGAAAGTATTTGTTGCCGTATTAACGAACCCGGCCA 300

Seq_1 301 CATATAAATTATTTTGCTCAATAATCAGACGAAGATGGTCAAAACGCGCCTGATAGACAT 360

||||||||||||||||||||||||||||||||||||||||||||||||||||||||||||

Seq_2 301 CATATAAATTATTTTGCTCAATAATCAGACGAAGATGGTCAAAACGCGCCTGATAGACAT 360

Seq_1 361 CAAGCCCTCGTATATCCACAGCAAAATAACTGCCCGGTGGGGTGTGGTTAATAACAGACA 420

||||||||||||||||||||||||||||||||||||||||||||||||||||||||||||

Seq_2 361 CAAGCCCTCGTATATCCACAGCAAAATAACTGCCCGGTGGGGTGTGGTTAATAACAGACA 420

Seq_1 421 CCGATGTGGTCCCCTGAGATATATGTTCAAGAGGGGTCGATATCTCTGTCCGTATACTAT 480

||||||||||||||||||||||||||||||||||||||||||||||||||||||||||||

Seq_2 421 CCGATGTGGTCCCCTGAGATATATGTTCAAGAGGGGTCGATATCTCTGTCCGTATACTAT 480

Seq_1 481 TTAACGAAGAGACATAACTTTGTTGGGTCGAAAAGTCTATCGTAAACT 528

||||||||||||||||||||||||||||||||||||||||||||||||

Seq_2 481 TTAACGAAGAGACATAACTTTGTTGGGTCGAAAAGTCTATCGTAAACT 528

Seq_3: gene encoding the B chain of the Stx2a purified in Bologna (Italy)

Seq_4: gene encoding the B chain of the Stx2a purified in Innsbruck (Austria)

Similarity: 197/198 (99.49 %)

Seq_3 1 GGTACTGGATTTGATTGTGACAGTGATTCCTGTCAGCTGAGCACTTTGCAGTAACGGTTG 60

||||||||||||||||||||||||||||||||||| ||||||||||||||||||||||||

Seq_4 1 GGTACTGGATTTGATTGTGACAGTGATTCCTGTCAACTGAGCACTTTGCAGTAACGGTTG 60

Seq_3 61 CAGATTCCAGCGACTGGTCCAGTATTCTTTCCCGTCAACCTTCACTGTAAATGTGTCATC 120

||||||||||||||||||||||||||||||||||||||||||||||||||||||||||||

Seq_4 61 CAGATTCCAGCGACTGGTCCAGTATTCTTTCCCGTCAACCTTCACTGTAAATGTGTCATC 120

Seq_3 121 CTCATTATACTTGGAAAACTCAATTTTACCTTTAGCACAATCCGCCGCCATTGCATTAAC 180

||||||||||||||||||||||||||||||||||||||||||||||||||||||||||||

Seq_4 121 CTCATTATACTTGGAAAACTCAATTTTACCTTTAGCACAATCCGCCGCCATTGCATTAAC 180

Seq_3 181 AGAAGCTAATGCAAATAA 198

||||||||||||||||||

Seq_4 181 AGAAGCTAATGCAAATAA 198

**FIGURE S1** Sequence of the genes encoding the A and the B subunits of Stx2a purified in Innsbruck (*E. coli* strain R82 (pJES) 120DH5α) and in Bologna (*E. coli* C600 (933W).


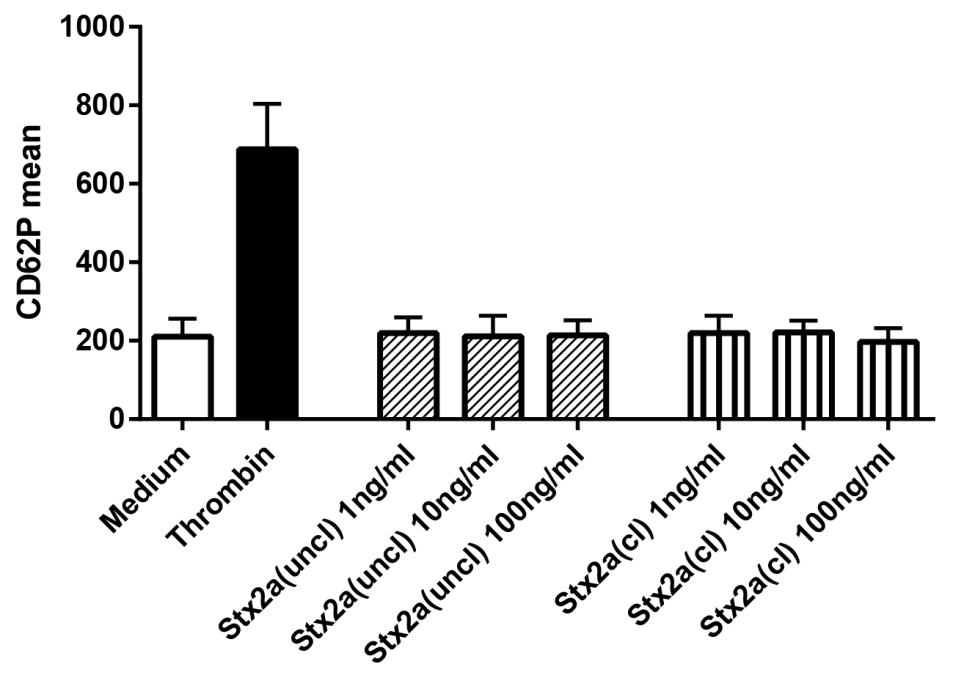


**FIGURE S2** Lack of P-selectin (CD62P) expression as a measure of platelet activation in PRP in the presence of Stx2a(cl) and Stx2a(uncl). The vertical axis represents the mean fluorescence intensity. FACS analysis of platelet activation was performed by incubation of PRP with medium, thrombin (positive control) and various concentrations of Stx2a(cl) or Stx2a(uncl). The results are the means ± S.D. of five separate experiments.

**FIGURE S3** Effect of monoclonal antibody to Stx2a on the inhibition of Raji cells protein synthesis by Stx2a(uncl) and trypsin-cleaved Stx2a. Raji cells were treated 3 h with 2.5 pM toxins in the absence or in the presence of the monoclonal antibody (10 µg) used to detect Stx2a bound to neutrophils (Stx2-BB12) then protein synthesis was measured in the presence of labeled leucine as described under Experimental procedures. *** p < .001
